# Case report: Rosai-Dorfman disease with rare extranodal lesions in the pelvis, heart, liver and skin

**DOI:** 10.3389/fonc.2022.1083500

**Published:** 2023-01-04

**Authors:** Misaki Yoshida, Takeshi Zoshima, Satoshi Hara, Yoshinori Takahashi, Ryo Nishioka, Kiyoaki Ito, Ichiro Mizuhima, Dai Inoue, Satoko Nakada, Mitsuhiro Kawano

**Affiliations:** ^1^ Department of Rheumatology, Kanazawa University Graduate School of Medical Sciences, Kanazawa, Japan; ^2^ Department of Radiology, Kanazawa University Graduate School of Medical Sciences, Kanazawa, Japan; ^3^ Department of Diagnostic Pathology, Kanazawa University Hospital, Kanazawa, Japan

**Keywords:** Rosai-Dorfman disease, histiocytosis, extranodal lesion, cervical lymphadenopathy, fever of unknown origin

## Abstract

Rosai-Dorfman disease (RDD), a rare form of non-Langerhans cell histiocytosis, can involve systemic extranodal lesions. Skin lesions are the most common, whereas intrapelvic, cardiac, and hepatic lesions are infrequent. The present study describes a 74-year-old woman with multiple extranodal lesions in the pelvis, heart, liver, and skin that were successfully treated with glucocorticoid therapy. She had experienced fever and persistent inflammation without cervical lymphadenopathy for several months and ^18^F-fluorodeoxyglucose (FDG) positron emission tomography (PET) showed abnormal FDG uptake in the left cheek; cervical, axillary, inguinal lymph nodes; periatrium; and pelvis. She was diagnosed with RDD based on skin and pelvic biopsies. Although this was an atypical case without bilateral cervical lymphadenopathy, the FDG-PET detection of inflammatory lesions led to selection of suitable biopsy sites, and pathological examination led to a correct diagnosis. Findings in this patient indicate that RDD can present with an atypical distribution of infrequent extranodal lesions, with attention required to prevent a delayed diagnosis.

## Introduction

Rosai-Dorfman disease (RDD) is a non-Langerhans cell histiocytosis characterized by bilateral cervical lymphadenopathy, fever, and an inflammatory response. It is rare, with a prevalence of 1 in 200,000. The mean age at onset is between the 20s and 30s, although some patients have been reported to develop RDD in their 70s. RDD is more common among people of African descent while the skin lesions are more common among Asian people (1). RDD has also been associated with rheumatic diseases and malignancies (1). Furthermore, fever or inflammation of unknown origin could be the first manifestation of RDD (2, 3). Pathological confirmation is required for the diagnosis of RDD, with this disease being characterized by the proliferation of histiocytes that are positive for S-100 protein and CD68 and negative for CD1a, as well as emperipolesis (4). Generally, RDD patients with only skin or lymph node lesions do not require treatment, as about 50% experience spontaneous remission. Surgical resection is the treatment of choice for patients with a single lesion. However, patients with severe conditions, disseminated extranodal lesions, or refractory disease are treated with immunosuppressive drugs, such as glucocorticoids.

Extranodal lesions have been reported in 43% of patients with RDD. These lesions can develop in almost all organs and adversely affect patient prognosis. About 67% of patients have only extranodal lesions (5, 6), whereas approximately 20% have lesions in multiple organs. The number of damaged organs correlates with patient prognosis (4). The most common extranodal sites include the skin (10–52%), bones (5–25%), head and neck (11–22%), kidneys (9%), and central nervous system (5–8%) (4, 6). Lesions in the heart and pelvis, however, are uncommon, and, to our knowledge, there have been no reports of their simultaneous development.

The present study describes an elderly woman who was diagnosed with RDD and had multiple extranodal lesions, including in the heart and pelvis, without bilateral cervical lymphadenopathy, a typical lesion of RDD. She had experienced inflammation of unknown origin for several months and was finally diagnosed with RDD with heart, pelvis, skin, and liver lesions. She was successfully treated with glucocorticoid. Although this was an atypical case without bilateral cervical lymphadenopathy, the detection of inflammatory lesions using ^18^F-fluorodeoxyglucose (FDG)-positron emission tomography (PET) led to the final diagnosis.

## Case report

**Figure 1:**
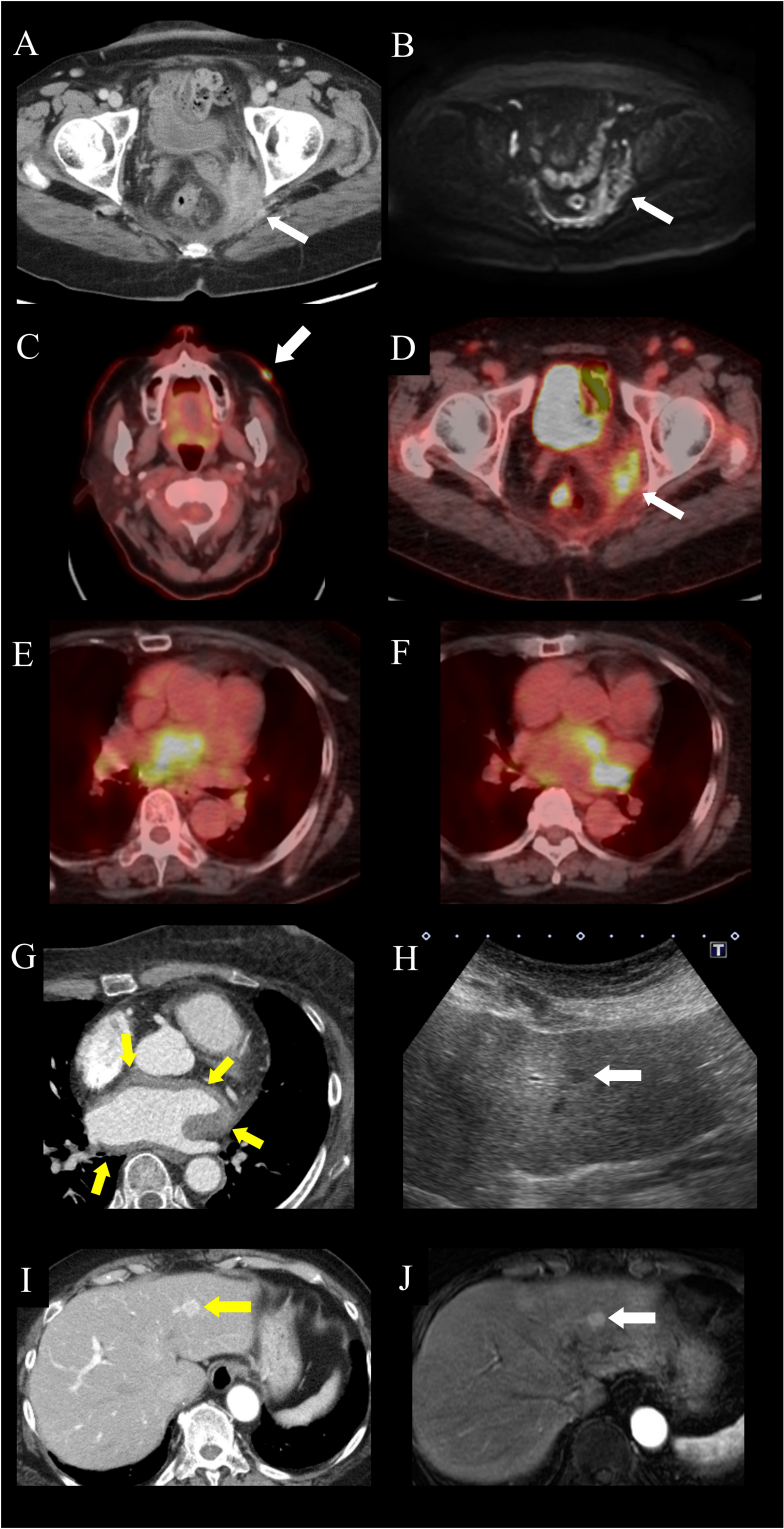
Images of extranodal lesions in the patient with Rosai-Dorfman disease. **(A, B)** Pelvic lesion. **(A)** Computed tomography (CT) scan showing an intra-pelvic mass enhanced predominantly in the peripheral zone. **(B)** Magnetic resonance imaging (MRI), of the intra-pelvic mass, showing heterogeneous high-intensity signals on diffusion-weighted imaging, accompanied by peritoneal thickening. **(C–F)** 18F-fluorodeoxyglucose-positron emission tomography (FDG-PET)-CT of the **(C)** facial skin lesion (arrow), with a maximum standardized uptake value (SUVmax) of 10.7, **(D)** pelvic lesion (arrow), with an SUVmax of 6.2, and **(E, F)** peri-atrial lesion spreading from the interatrial septum to the left atrium, with an SUVmax of 25. **(G)** Electrocardiography-gated contrast-enhanced CT, showing homogeneous wall thickening around the left atrium (arrows). **(H–J)** Hepatic lesion. **(H)** Ultrasonography, showing a homogeneous hypoechoic nodule (arrow). **(I)** Contrast-enhanced CT imaging, showing a hypervascular nodule in the left lateral hepatic segment. (arrow). **(J)** Gadolinium ethoxybenzyl diethylenetriamine pentaacetic acid-enhanced MRI, showing a homogeneously enhanced nodule in the arterial phase (arrow).

A 74-year-old woman was admitted to our hospital for closer examination of chronic inflammation. She had been treated for hypertension and type 2 diabetes for 16 years. Four years prior to hospitalization, she experienced a slight elevation of serum C-reactive protein (CRP) concentration, to around 0.5 mg/dL, but she was asymptomatic. Two years prior, she developed lower leg edema, which gradually worsened. One year previously, she developed puffy fingers and abnormal nailfold capillaries, and was positive for serum anti-centromere antibody but not Raynaud’s phenomenon. She was suspected of having systemic sclerosis but did not fulfill the classification criteria (7). Ten months prior, erythema developed on her face and limbs. A skin biopsy of the left lower leg revealed infiltration of inflammatory cells, mainly histiocytes, in the superficial intradermal area and infiltration of small lymphocytes and plasma cells in the perivascular area. Although the cause was not identified, the skin lesions improved following application of topical glucocorticoids. Nine months previously, she had developed dyspnea on exertion. Although a chest X-ray revealed cardiac dilatation, there was no evidence of pulmonary congestion. Treadmill exercise electrocardiogram showed transient atrial fibrillation. Chest computed tomography (CT) revealed pleural effusion, whereas echocardiography showed no asynergy. She was suspected of having mild congestive heart failure caused by paroxysmal atrial fibrillation. Her dyspnea improved after treatment with bisoprolol, apixaban, furosemide, and spironolactone. Eight months prior, her body temperature was 37°C and her serum CRP level was elevated to 4.78 mg/dL. Systemic CT showed a contrast-enhanced intra-pelvic mass with irregular margins, along with peripheral enhancement (**Figure 1A**), but no other lesions in the heart, liver, or lymph nodes. Magnetic resonance imaging (MRI) showed an intra-pelvic mass with low-intensity signals on T2-weighted images and heterogeneous high-intensity signals on diffusion-weighted images (**Figure 1B**). Intra-pelvic thickening of the peritoneum was also observed. Cervical cytology revealed the absence of malignant cells. Because a pelvic abscess was suspected, antibiotics were prescribed, but there were no improvements in serum CRP levels or the size of the pelvic lesion. Her serum CRP level persisted at around 2 mg/dL, accompanied by a weight loss of 5 kg in 6 months. One month prior to admission, a white keratotic nodule appeared on her left cheek. FDG-PET-CT revealed abnormal FDG uptake in the left cheek; cervical, axillary, and inguinal lymph nodes; periatrium, colon, and pelvis (**Figures 1C–F**). These PET-CT results suggested that malignant lymphoma was highly likely.

**Figure 2:**
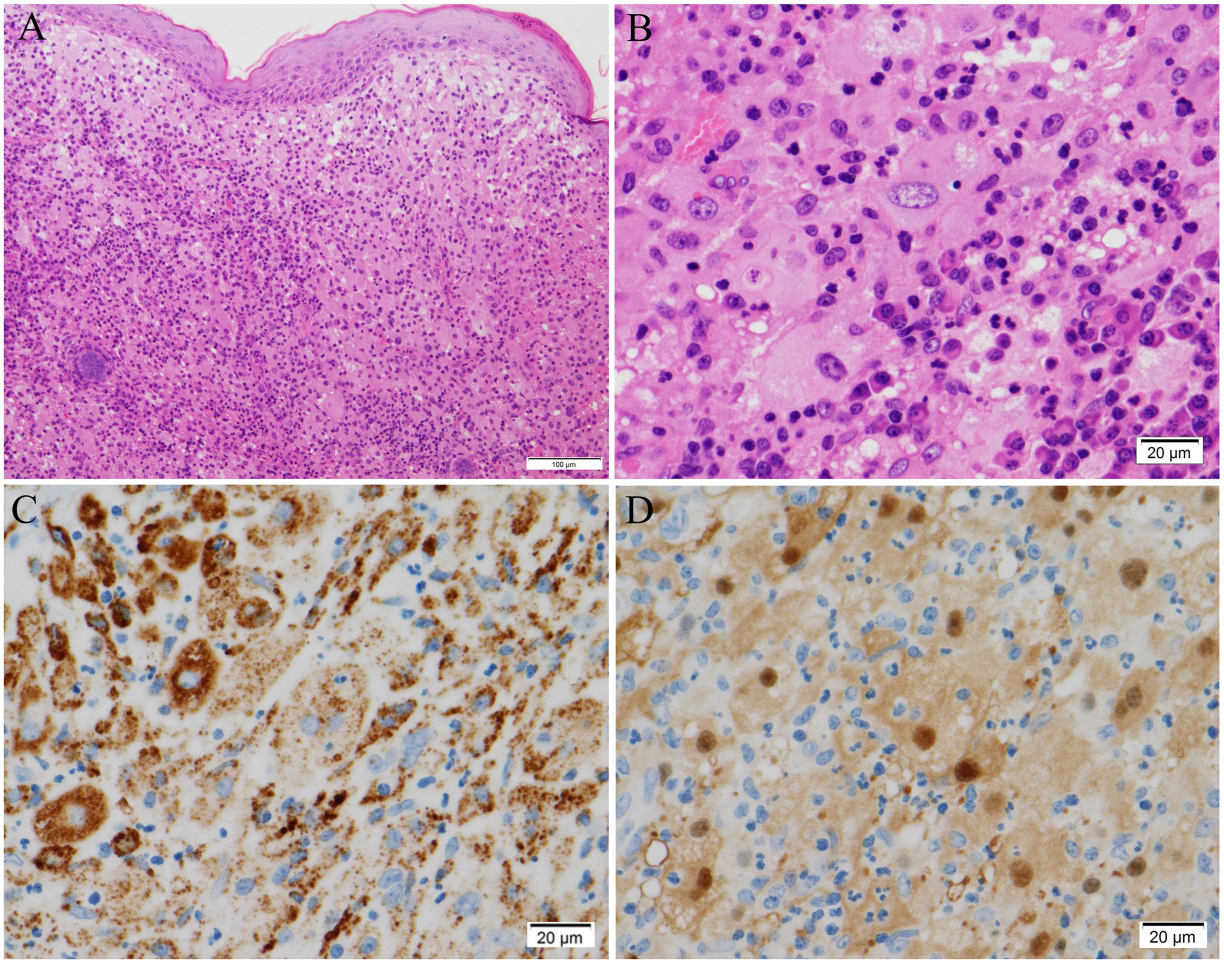
Histological findings of the facial skin biopsy. **(A, B)** Hematoxylin and eosin staining, showing **(A)** large round cells aggregated diffusely in the upper dermis immediately beneath the epidermis, and **(B)** emperipolesis, in which lymphocytes and neutrophils are engulfed. **(C, D)** Immunohistochemical analysis, showing that the large round cells were positive for **(C)** CD68 and **(D)** S-100 protein. Original magnifications: × 100 **(A)**, × 400 **(B–D)**. Scale bars: 100 μm **(A)** and 20 μm **(B–D)**.

On admission, cervical lymphadenopathy was not observed. A murmur (Levine II/VI) was heard at the left second sternal border. Abdominal examination yielded normal results. Bilateral lower leg edema was detected. Facial erythema had disappeared, and the erythema on both legs had changed to brownish or yellowish pigmentation. Laboratory examination revealed elevated levels of serum CRP (2.72 mg/dL) and soluble interleukin-2 receptor (965 U/mL). The patient was positive for anti-centromere antibodies, but there was no evidence of other rheumatic diseases including IgG4-related disease or infections (**Table 1**). Echocardiography showed hyperechoic thickening at the interatrial septum and irregular thickening with heterogeneous echoic brightness at the left atrial wall behind the aorta. Electrocardiography-gated contrast-enhanced CT revealed homogeneous wall thickening around the left atrium (**Figure 1G**). Abdominal ultrasonography showed a homogeneous hypoechoic nodule (**Figure 1H**). Contrast-enhanced CT and gadolinium ethoxybenzyl diethylenetriamine pentaacetic acid (Gd-EOB-DTPA)-enhanced MRI demonstrated a hyper vascular nodule approximately 10 mm in diameter in the left lateral hepatic segment (**Figures 1I, J**). These lesions had not been detected in images acquired 6 months before hospitalization. Lower gastrointestinal endoscopy did not reveal any lesions. Skin biopsy of the left cheek showed diffuse aggregations of large round histiocytes in the upper dermis just beneath the epidermis. Some histiocytes engulfed lymphocytes and neutrophils, a finding compatible with emperipolesis. Immunohistochemistry revealed that these histiocytes were positive for CD68 and S-100 protein (**Figure 2**). There was no evidence of histiocytosis other than RDD. In addition, malignant cells were not observed and there were no indications of clonal proliferation of kappa and lambda chains. The ratio of IgG4-positive cells to CD138-positive cells was approximately 10%. CT-guided biopsy of the pelvic mass (**Supplemental Figure 1**) and re-examination of the skin biopsy of the leg obtained before hospitalization showed similar findings. Next-generation sequencing analysis for mutations in the MAPK pathway was not performed. However, based on these histological findings, the patient was diagnosed with RDD involving the pelvis, skin, heart, liver, and lymph nodes.

**Table 1:** Laboratory Data on Admission.

	Value	Normal range
Urinalysis		
Protein	(−)	(−)
Occult blood	(−)	(−)
Blood count		
White blood cells (/μL)	8,260	3,300-8,800
Neutrophil (%)	80.7	
Eosinophil (%)	1.6	
Lymphocyte (%)	13.1	
Hemoglobin (g/dL)	9.5	13.5-17.0
Platelet (/μL)	32.3×10^4^	13.0-35.0×10^4^
Erythrocyte sedimentation rate (mm/h)	54	3-15
Serum chemistry		
BUN (mg/dL)	17	8.0-20.0
Cr (mg/dL)	0.79	0.60-1.00
Na (mEq/L)	141	135-149
K (mEq/L)	3.9	3.5-4.9
Cl (mEq/L)	105	96-108
AST (IU/L)	18	13-33
ALT (IU/L)	9	8-42
LDH (IU/L)	167	119-229
Total protein (g/dL)	7	6.7-8.3
Albumin (g/dL)	3.5	4.0-5.0
HbA1c(%)	7.5	5.6-5.9
Immunological findings		
CRP (mg/dL)	2.72	0-0.3
IgG (mg/dL)	2,098	870-1,700
IgG4 (mg/dL)	90.6	4.8-105
IgA (mg/dL)	217	110-410
IgM (mg/dL)	85	46-260
C3 (mg/dL)	111	65-135
C4 (mg/dL)	31	13-35
CH50 (U/mL)	69	25.0-48.0
Soluble interleukin-2 receptor (U/mL)	965	157-474
Anti-nuclear antibody	×80 (AC, H)	<40
Anti-RNP antibody (U/mL)	(−)	(−)
Anti-SS-A antibody (U/mL)	<10.0	<10.0
Anti-Scl-70 antibody (U/mL)	<10.0	<10.0
Anti-centromere antibody (U/mL)	24.8	<10.0
Anti-RNA polymerase III antibody	(−)	(−)
Anti-ARS antibody	(−)	(−)
MPO-ANCA (IU/mL)	<1.0	<1.0
PR3-ANCA (IU/mL)	<1.0	<1.0
NT-proBNP (pg/mL)	3,597	<125
HBsAg (IU/mL)	<0.001	<0.05
HBsAb (IU/mL)	<0.1	<10
HBcAb	(−)	(−)
HCVAb	(−)	(−)
Interferon-gamma release assay	(−)	(−)
β-D-glucan (pg/mL)	<6.0	<11.0
Cytomegalovirus antigen	(−)	(−)

**Figure 3:**
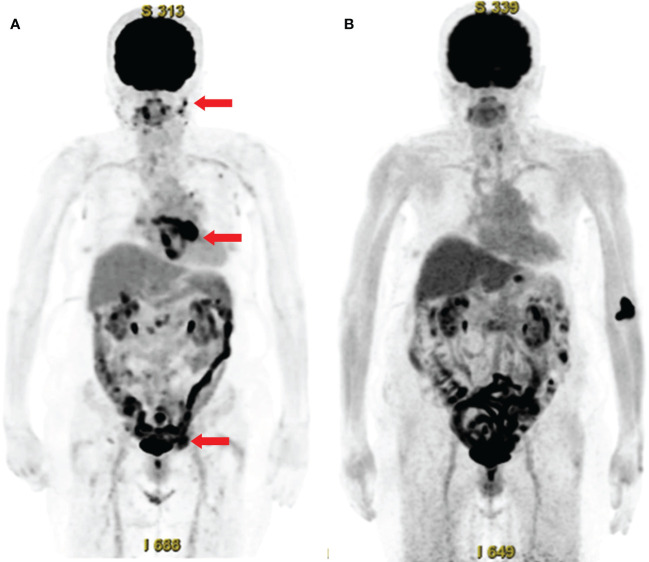
Whole-body ^18^F-fluorodeoxyglucose (FDG)-positron emission tomography with computed tomography (PET-CT) before and after treatment. **(A)** FDG-PET-CT before treatment, showing abnormal FDG uptake in the facial skin, peri-atrial, pelvic (arrows), cervical, axillary, and inguinal lymph nodes, and colon. **(B)** Follow-up FDG-PET-CT after treatment, showing the disappearance of abnormal FDG uptake.

The patient was started on treatment with 50 mg/day prednisolone (0.75 mg/kg/day). Nine days later, her serum CRP levels normalized. Fourteen days later, however, she developed an arterial flutter, which was successfully treated with electrical cardioversion, bisoprolol, and verapamil. A follow-up PET-CT scan six months later showed significantly reduced FDG uptake by the above-mentioned lesions (**Figure 3**). Furthermore, abdominal ultrasonography revealed that the liver nodule had disappeared.

## Discussion

This report describes an elderly patient who presented with RDD involving the pelvis, heart, liver, skin, and lymph nodes. This patient was difficult to diagnose because she only had extranodal lesions, such as pelvic and cardiac lesions, which are infrequently associated with RDD, and showed no evidence of bilateral cervical lymphadenopathy.

Pelvic lesions are rare in patients with RDD and have been described only in case reports (8). CT findings are non-specific, but invasive masses have been reported to surround the kidneys, ureters, and blood vessels, as has soft tissue with lymphadenopathy (9). The incidence rate of intraperitoneal lesions, including those in the pelvis, is 4% (8). The onset of intraperitoneal lesions occurs during the seventh and eighth decades of life (8). When only intraperitoneal lesions are detected, it is important to exclude neoplastic lesions other than RDD (9). In this case, pelvic pathology was consistent with RDD, with these lesions considered pelvic manifestations of RDD.

Cardiac lesions are also rare extranodal lesions of RDD, being observed in only about 0.1% of patients with RDD (10). Cardiac lesions have been classified into three types: intracardiac masses, pericardial/epicardial involvement, and pulmonary arterial involvement (11). An analysis of 15 patients showed that the mean age of these patients was 49.5 years (range, 22–79 years) and that intracardiac masses were the most common cardiac lesions, being observed in nine (60%) of these 15 patients (11). In addition, 8 patients (53%) had no lesions other than cardiac lesions. The most common symptoms were chest pain and shortness of breath, followed by palpitations and edema. Nine patients underwent surgical resection, including six with intracardiac masses and three with pulmonary artery masses. Glucocorticoids were administered to three patients, one each with an intracardiac mass, pericardial/epicardial lesion, and pulmonary arterial mass. Although the observation period was not reported, all patients treated surgically were alive at the time of last follow-up, suggesting that surgical removal of these lesions is associated with a favorable prognosis. However, four (27%) of these patients died. Although the present patient did not undergo cardiac biopsy, her cardiac lesion was consistent with RDD, probably because the cardiac lesion improved along with her other lesions, following treatment with glucocorticoid.

Hepatic lesions are infrequent in patients with RDD patients, being reported in only 1–5% (4, 6). These patients show single or multiple hepatic nodules, or hepatomegaly (8, 9, 12). One report described 11 patients with gastrointestinal lesions, including five with hepatic lesions (12). Four of the latter had other extranodal lesions, including two with cardiac lesions, similar to the present patient.

Recently, RDD has been diagnosed at ages older than previously reported. A report describing 423 RDD patients published in 1990 showed that most cases of RDD developed in childhood, with an average age of onset of 20–30 years; age at diagnosis was not reported (4). In contrast, a report of 64 patients published in 2020 showed that the median age at diagnosis was 50 years (interquartile range [IQR] 2–79 years), with the median period from onset to diagnosis being 7 months (IQR 0–128 months) (6). Moreover, a report of 10 patients at a single facility published in 2019 showed that the mean age was 56 years (IQR 20–81 years) (13). The present patient was diagnosed with RDD at age 74 years.

The diagnosis of RDD may be delayed because of its low prevalence and late recognition. RDD is a rare disease that is often undiagnosed at early stages (14). There are many differential diagnoses, including infections with, for example, acid-fast bacilli and fungi; and malignant diseases, including malignant lymphoma; Erdheim-Chester disease; and IgG4-related disease. Each extranodal lesion has a differential diagnosis (1). Furthermore, emperipolesis may occur in other conditions such as malignant lymphoma, leukemia, myelodysplasia, and myeloma. For diagnosis with RDD, immunohistochemical analyses including positive for S-100 and CD68 and negative for CD1a are important (1, 4). One of the typical findings of RDD is cervical lymphadenopathy; however, many recent reports have described patients with RDD without lymphadenopathy (6). Moreover, some patients with RDD, such as the present patient, present with fever or inflammation of unknown origin (2, 3).

FDG-PET-CT has been shown useful for the identification of sites that can be biopsied and for the evaluation of responses to treatment (1). The present patient underwent biopsies of areas of the skin and pelvic lesions with abnormal FDG uptake. However, the cardiac and hepatic lesions were not biopsied because of the risk of hemorrhage when direct oral anticoagulants were administered. Because these cardiac and hepatic lesions appeared at the same time as the other RDD lesions and their responses to glucocorticoid treatment were similar, they were diagnosed as RDD-associated lesions. In addition, diagnosis may be delayed in patients with atypical distribution of infrequent lesions, such as the present patient.

In conclusion, this report describes a rare case of RDD in an elderly patient with extranodal lesions in the pelvis, heart, liver, and skin. Although this presentation was atypical without bilateral cervical lymphadenopathy, detection of inflammatory lesions using FDG-PET led to the selection of suitable biopsy sites, with subsequent pathological examination resulting in a correct diagnosis. These findings indicate that RDD may present with an atypical distribution of infrequent extranodal lesions, with attention being required to prevent a delayed diagnosis.

## Data availability statement

The raw data supporting the conclusions of this article will be made available by the authors, without undue reservation.

## Ethics statement

The studies involving human participants were reviewed and approved by Kanzawa University Hospital. The patients/participants provided their written informed consent to participate in this study.

## Author contributions

MY, TZ, and MK wrote the draft and revised it. SH, YT, RN, KI, IM, DI, and SN provided some important advice for the draft. All authors contributed to the article and approved the submitted version.

## Supplementary material

Supplementary Figure 1 Histological findings of the pelvic lesion. **(A, B)** Hematoxylin and eosin staining revealed lymphoplasmacytic infiltration in fat tissue and scattered histiocytes with emperipolesis. **(C)** Immunohistochemical analysis for S-100 protein showing histiocytes with emperipolesis. Original magnifications: × 100 (A), × 400 **(B, C)**.
