# Supplementary figures and images for: Case report: Rosai-Dorfman disease with rare extranodal lesions in the pelvis, heart, liver and skin
Source: Front Oncol. 2023 Jan 4;12:1083500. doi: 10.3389/fonc.2022.1083500 (PMC9846742; doi:10.3389/fonc.2022.1083500)

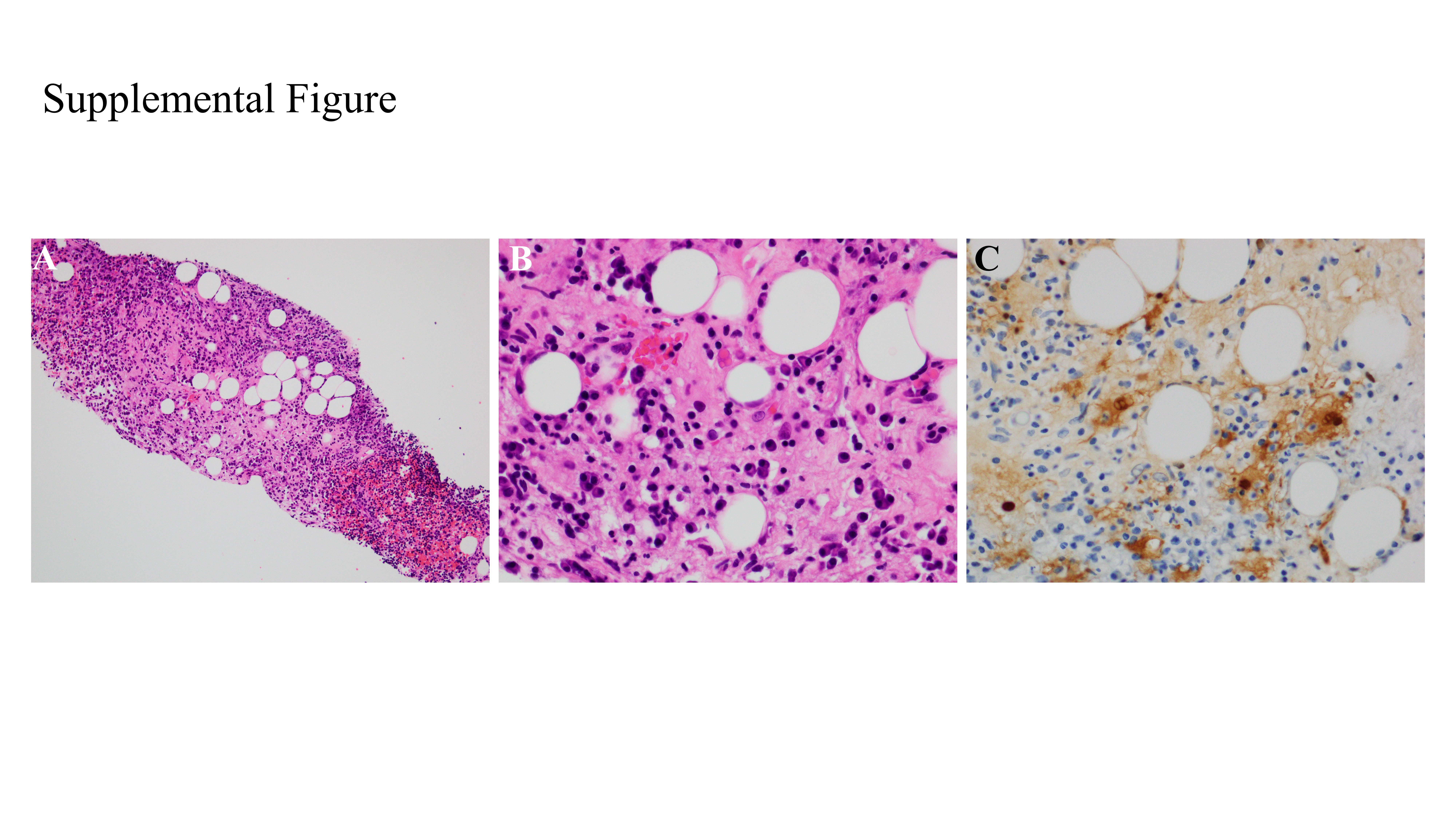

Supplement: Supplementary Figure 1 — Histological findings of the pelvic lesion. (A, B) Hematoxylin and eosin staining revealed lymphoplasmacytic infiltration in fat tissue and scattered histiocytes with emperipolesis. (C) Immunohistochemical analysis for S-100 protein showing histiocytes with emperipolesis. Original magnifications: × 100 (A), × 400 (B, C). [file Image_1.jpeg]
